# Frequency and determinants of vitamin D deficiency among premenopausal and postmenopausal women in Karachi Pakistan

**DOI:** 10.1186/s12905-021-01339-9

**Published:** 2021-05-10

**Authors:** Uzma Shamsi, Iqbal Azam, Azra Shamsi, Dua Shamsi, David Callen

**Affiliations:** 1 School of Medicine, Adelaide Medical School, University of Adelaide, Adelaide, Australia; 2 Department of Community Health Sciences, Aga Khan University, Karachi, Pakistan; 3 Department of Gynecology and Obstetrics, Combined Military Hospital, Karachi, Pakistan; 4 Division of Data Science, University of California, Berkeley, USA

**Keywords:** Vitamin D deficiency, Socioeconomic status, Postmenopausal, Premenopausal

## Abstract

**Background:**

Vitamin D deficiency is becoming a serious public health problem, even in sun-drenched cities like Karachi, Pakistan. We investigated the prevalence of vitamin D deficiency and its association with sociodemographic characteristics, anthropometric measures, and lifestyle factors among premenopausal and postmenopausal women (n = 784).

**Methods:**

Face-to-face interviews were conducted to collect information and serum concentrations of 25-hydroxyvitamin D were measured after the interviews.

**Results:**

A total of 57% of women were vitamin D deficient with higher vitamin D deficiency found among premenopausal women (64.7%) compared to postmenopausal women (49%). The median serum concentrations of 25-hydroxyvitamin D (IQR) were 16.7 ng/ml (IQR 9.8–30.0). Factors associated with vitamin D deficiency were lower socioeconomic status (OR 2.00; 95% CI 1.15–3.48), younger age with highest vitamin D deficiency found in < 35 years of age group (OR 3.11; 95% CI 1.76–5.51), and winter season (OR 1.51, 95% CI 1.07–2.15) after adjusting for multiple confounders. The use of vitamin D supplement (OR 0.59, 95% CI 0.38–0.92) and vigorous exercise (OR 0.20, 95% CI 0.05–0.80) were protective against vitamin D deficiency.

**Conclusions:**

The study shows a high prevalence of vitamin D deficiency, with detrimental health effects, among younger women belonging to lower socioeconomic status and during the winter season. The use of vitamin D supplements and vigorous exercise were protective measures. Public health campaigns are needed for education and awareness about vitamin D deficiency to improve vitamin D status for younger women living in poor environments.

**Supplementary Information:**

The online version contains supplementary material available at 10.1186/s12905-021-01339-9.

## Background

Vitamin D deficiency VDD (< 20 ng/ml) is becoming increasingly common in majority of population worldwide. There is also a lot of research interest in studies assessing the factors causing VDD and role of VDD in many diseases including the Covid 19 infection [1, 2]. VDD is an important public health problem in Pakistan too. According to a study in Hyderabad, VDD and vitamin D insufficiency combined was 78.3% among 1244 healthy individuals [3]. In another study at Ayub Teaching Hospital Abbottabad, high VDD was reported among 202 patients (63.4%) with complaints of generalized body aches [4]. A study conducted among healthy women of child-bearing age in Lahore reported that VDD was 73%. Factors associated with VDD were lesser sun exposure, illiteracy, winter season during sampling and no usage of multivitamins. A cross-sectional population survey among 300 adult population of Karachi showed median concentration of serum vitamin D as 18.8 ng/ml with high prevalence of VDD [5]. VDD was reported as 90.1% among 305 premenopausal females in another cross-sectional study in Karachi, Pakistan. Factors associated with VDD were age, city of residence in downtown and suburbs, and type of housing [6]. In a recent study among 221 medical students of a medical school, VDD was found in 89.1% and factors associated with VDD were limited sun exposure and attire consisting of full length of sleeves outside [7].

There is a lot of variation in the vitamin D status of women living in Karachi in different studies, most of which consisted of smaller sample sizes and only few potential factors responsible for VDD were assessed. Therefore, the objective of the study was to assess the frequency of VDD and investigate all the factors associated with VDD among women of both premenopausal and postmenopausal status in Karachi Pakistan.

## Methods

### Study population

We extracted data of 784 women, who were in the control group and had complete information on potential predictors of VDD and serum 25(OH)D concentrations, from the case control study (the details of which are mentioned in a recently published article) [8]. Briefly, two major hospitals were selected to have a representative sample in Karachi, which is the 5th most populous city in the world: Aga Khan University Hospital (AKUH) is a private teaching hospital in Pakistan and ISO 9002 certified. Karachi Institute of Radiation and Nuclear Medicine Hospital (KIRAN), is a public hospital, which also provides free diagnosis and treatment facilities to more than 75% of its patients, who come from all over the country. All the women were interviewed attending in- and out-patient services for general medical, and surgical departments. Exclusion criteria were history of any cancer, parathyroid, renal or liver disease, chronic malabsorption syndromes or any known or suspected drug that may alter vitamin D metabolism.

Face to face interviews were conducted and information was collected about sociodemographic factors, clinical and reproductive history, sun exposure, use of vitamin D supplementation, and anthropometric measurement. Age was categorized into < 35, 35–45, 46–54, 55 years. Education was categorized as < 8, 8–12, or > 12 years of education. Socio-economic (SES) factors included multiple variables like education, place and type of residence, home ownership, number of rooms, total household members, total household monthly income and crowding index (the number of household members divided by number of rooms) A composite variable for SES was calculated by factor analysis of all these important variables. Finally, SES was categorized into upper, middle and lower status [8].

#### Sun exposure measurement questionnaire

The validated Long-Term Sun Exposure Measurement Questionnaire (LT SEM-Q) was used to assess sun exposure among all women [9]. This questionnaire assessed sunlight exposure, duration of sun exposure, skin tone, use of sunscreens and sun avoidance behaviour. The time spent outdoors between 10 am and 4 pm was asked to calculate approximate amount of time in minutes per week that women were exposed to UVB radiations*.* Details of weights given to sun exposure variables are in the Additional File 1: Table 1. The final scoring algorithm of sun exposure score in summers and winters was created by multiplying the time (minutes) spent in the sun by the proportions of the different variables. The skin tone of the women was assessed using a shade card (10 skin tones (1–10)) by matching shade of the skin on forehead with the shade on the card, according to LT SEM-Q.

#### Vitamin D supplements

Use of vitamin intake was asked in detail including frequency, duration and form of intake as injectable, oral drops or capsules apart from intake as yes vs. no. The use of Vitamin D supplement including multivitamins was assessed by asking participants if they had been taking Vitamin D supplements regularly, occasionally or not at all in their lifetime. Information was also obtained about form and mode of administration (injections, oral tablets/capsules or ampoule drops). Herbal medications which possibly increase Vitamin D inactivation was also taken into consideration. In order to determine if there were possible drug interactions, participants were also asked whether they had taken anti-hypertensive, anti-epileptic, anti-inflammatories and endocrine drugs.

#### Measurement of serum 25(OH)D concentrations

Venous blood samples (2.5 ml) were taken from all participants and collected in yellow topped gel tubes at the end of the interview. Serum 25(OH)D concentrations were measured by a kit from DiaMetra SRL (Via Garibaldi, 18-20090 Segrate Italy), using solid phase enzyme-linked immunoassay (ELISA). The cut-off used to define VDD was according to the Parathyroid Hormone (PTH) levels [10].Vitamin D deficiency was defined as a serum 25(OH)D concentrations < 20 ng/ml. Season of blood collection (winter/ spring vs. summer /fall) was also recorded.

#### Vitamin D containing food items

There are limited major sources of vitamin D in the diet. Each participant was asked about following dietary intake of Vitamin D containing food items: milk, yogurt, lassi, eggs, fish (fatty or white fish), any fortified cereals, breads or other products. over the past year. Intake frequency was categorised into 7 groups ranging from “never” to “5–6 times per day” for these foods and beverages. The selected frequency category for each food item was converted to a daily intake. For example, a response of “one serving/week” was converted to 0.14 servings/day by dividing 1 by 7. Each participant was also asked about their average portion size/ common serving size of the food. A common serving (svg) size of food item or beverage was specified on the FFQ (e.g. 1 cup of milk or one egg,). The intake frequencies were multiplied by standard portion size to calculate servings per day of all the food items. However, consumption in grams per unit or macronutrient intakes or calories from each food was not calculated.

Physical activity was also measured and the International Physical Activity Questionnaire (IPAQ) index was used [11]. Physical activity was categorized into vigorous exercise like heavy lifting, digging, aerobics and moderate exercise like carrying light loads, mopping etc. that makes one breathe somewhat harder than normal and walking and women recalled the number of days and hours or minutes, they were engaged in physical activity of different intensities for at least ten minutes.

Other variables included BMI (calculated as weight in kilograms divided by the square of height in meters). Women were categorized into three groups according to the World Health Organization (WHO) cut off for BMI in Asian population [12]: a normal weight (18.5–24.9 kg/m^2^), being overweight (25.0–29.9 kg/m^2^) or obese (> 30 kg/m^2^).

### Statistical analysis

Mean and standard deviations are reported for continuous variables and frequencies and percentages for categorical variables to illustrate the differences in vitamin D concentrations between premenopausal and postmenopausal women. Univariable analysis was done to calculate unadjusted OR and 95% CI. Variables < 0.25 and those variables with biological plausibility were included and entered in multivariable logistic regression in a step-wise fashion to calculate adjusted ORs and 95% CIs. All reported *p* values are based on two-sided tests. Statistical analysis of the data was carried out using the SPSS package for Windows 22.0 (SPSS, IBM, Armonk, NY, USA).

## Results

The median concentration of serum 25(OH)D was 16.7 ng/ml (IQR 9.8–30.0). There was a high prevalence of VDD among both premenopausal and postmenopausal women. Individual 25(OH)D concentrations ranged from 0.3 ng/ml to 165.5 ng/ml. The median 25(OH)D concentration was lower among premenopausal women (13.9 ng/ml, IQR 8.0–25.2) compared to postmenopausal women (20.4 ng/ml, IQR 11.1–31.7).

Women were further categorized into four groups according to the different concentrations of 25(OH)D, defined as severely deficient (< 12 ng/ml), deficient (12–19 ng/ml), insufficient (20–30 ng/ml) and sufficient (> 30 ng/ml) (Table 1). Both severe vitamin D deficiency and deficiency was significantly more frequent in premenopausal women compared to postmenopausal women*.*

**Table 1:** Number and percentage of women for different serum 25(OH)D concentrations

Serum 25(OH)D (ng/ml)	Premenopausal women n (%)	Postmenopausal women n (%)	All women n (%)	*p* value*
Severely deficient (< 12)	124 (39.9)	78 (26.2)	202 (33.2)	< 0.001
Deficient (12–19)	77 (24.8)	68 (22.8)	145 (23.8)	
Insufficient (20–30)	52 (16.7)	61 (20.5)	113 (18.6)	
Sufficient (> 30)	58 (18.6)	91 (30.5)	149 (24.5)	

^*^p values generated from Chi-square test

Table 2 shows that VDD was associated with younger age, with highest VDD among women of < 35 years of age group (adjusted OR 3.11; 95% CI 1.76–5.51), and lower socioeconomic status (adjusted OR 2.00; 95% CI 1.15–3.48 after adjusting for multiple confounders (*p* < 0.0001). Use of vitamin D supplement (adjusted OR 0.59, 95% CI 0.38–0.92) and vigorous exercise (adjusted OR 0.20, 95% CI 0.05–0.80) were protective against VDD. As it was a hospital-based study, it was observed that a higher percentage (63%) of women took vitamin D supplements but fish intake was less frequent and nearly 40% women ate no fish at all. The detailed analysis of different types of vitamin D supplements and vitamin D containing food items is given in supplementary Tables 2 and 3.

**Table 2:** Socio-demographic and clinical characteristics of both premenopausal and postmenopausal women (n = 784) according to serum 25 (OH) D concentrations (ng/ml) in Karachi, Pakistan

Variables	Vitamin D ≥ 20	Vitamin D deficiency < 20	Crude OR (95% CI)	Adjusted OR (95% CI)
Age groups	n (%)	n (%)		
< 35	29 (10.9)	65 (18.7)	3.46 (2.00, 5.97)	3.11 (1.76, 5.51)
35–45	77 (28.9)	144 (41.4)	2.88 (1.88, 4.43)	2.66 (1.69, 4.19)
46–54	69 (25.9)	80 (23.0)	1.79 (1.13, 2.83)	1.72 (1.07, 2.77)
55 and above	91 (34.2)	59 (17.0)	Ref	Ref
Education				
< Grade8	49 (18.4)	68 (19.5)	1.07 (0.69, 1.65)	
Grades 8–12	87 (32.7)	111(31.9)	0.98 (0.68, 1.41)	
> Grade12	130 (48.9)	169 (48.6)	Ref	
Socioeconomic status				
Lower	61 (22.9)	52 (14.9)	2.62 (1.58, 4.34)	2.0 (1.15, 3.48)
Middle	158 (59.4)	191 (54.9)	1.42 (0.92, 2.17)	1.23 (0.77, 1.95)
Upper	47 (17.7)	105 (30.2)	Ref	Ref
Parity*				
Nullipara	34 (12.8)	59 (17.0)	0.82 (0.50, 1.350	
1 –3	136 (51.1)	152 (43.7)	0.64 (0.40, 1.04)	
> 3	96 (36.1)	137 (39.4)	Ref	
Menopausal status				
Postmenopausal	152 (58.0)	146 (43.0)	1.90 (1.38, 2.63)	
Premenopausal	110 (42.0)	201 (57.0)	Ref	
History of any comorbid				
Yes	161 (60.5)	168 (48.3)	0.61 (0.44, 0.84)	
No	105 (39.5)	180 (51.7)	Ref	
Season vitamin D				
Winter	102 (39.2)	167 (48.7)	1.47 (1.06, 2.04)	
Summer	158 (60.8)	176 (51.3)	Ref	
Body mass index^a^				
Normal	70 (27.9)	80 (25.1)	Ref	
Over weight	108 (43.0)	130 (40.8)	1.05 (0.69, 1.58)	
Obese	73 (29.1)	109 (34.2)	1.31(0.85, 2.02)	
Fish consumption				
No intake	107 (41.2)	136 (40.1)	1.14 (0.56, 2.29)	
< Than once a month	136 (52.3)	184 (54.3)	1.21 (0.61, 2.42)	
> Once/month	17 (6.5)	19 (5.6)	Ref	
Vitamin D supplements intake				
Yes	204 (76.7)	172 (49.4)	0.30 (0.19, 0.46)	0.59 (0.38, 0.92)
No	62 (23.3)	176 (50.6)	Ref	Ref

*OR* odds ratio; *CI* confidence interval

Parity* was restricted to women who ever had a full-term pregnancy (a pregnancy was considered as full-term if it resulted in a live birth or lasted 7 or more months)

^a^*BMI* body mass index; BMI was categorized according to the WHO classification for Asian as normal weight (< 18.5–24.9 kg/m^2^), overweight (25.0–29.9 kg/m^2^) or obese (> 30 kg/m^2^)

Reference: OR are compared to those women who had VDD. Adjusted for education, BMI, parity, skin tone, sunlight exposure, attire outside. Reference: OR are compared to those women who had no vitamin D deficiency

Table 3 shows lifestyle and sun exposure factors related to VDD. Moderate physical activity, consisting of mostly household activities, was most common among women compared to vigorous exercise and walking. Vigorous exercise > 3.5 hors/week, though it was less common among all women, but it was protective against **v**itamin D deficiency (adjusted OR 0.20, 95% CI 0.05–0.80). VDD was also associated with winter season (adjusted OR 1.51, 95% CI 1.07–2.15). The majority of the women had skin tone of 5 and 6 categorized as wheatish tone, however, skin tone was not associated with serum 25(OH)D concentrations.

**Table 3:** Lifestyle and sun exposure related factors associated with serum 25(OH)D concentrations (ng/ml) in Pakistani women

Categories	Vitamin D ≥ 20 n (%)	Vitamin D < 20 n (%)	Crude OR (95% CI)	Adjusted OR (95% CI)
Vigorous exercise				
< 3.5 h/week	17 (6.4)	7 (2.0)	0.82(0.29, 2.28)	0.63 (0.21, 1.86)
> 3.5 h/week	6 (2.3)	10 (2.9)	0.25 (0.07, 0.95)	0.20 (0.05, 0.80)
No exercise	243 (91.4)	331 (95.1)	Ref	Ref
Moderate exercise				
< 3.5 h/wk	32 (12.0)	58 (16.7)	0.59 (0.40, 0.87)	
> 3.5 h/wk	54 (20.3)	98 (28.2)	1.00 (0.58, 1.72)	
No exercise	180 (67.7)	192 (55.2)	Ref	
Walking				
< 3.5 h/wk	78 (29.3)	88 (25.3)	1.59(1.00, 2.53)	
> 3.5 h/wk	46 (17.3)	44 (12.6)	1.18 (0.71, 1.97)	
No exercise	142 (53.4)	216 (62.1)	Ref	
Head covered				
Yes	171 (64.3)	271 (78.1)	1.98 (1.39, 2.83)	
No	95 (35.7)	76 (21.9)	Ref	
Face covered				
Yes	39 (14.7)	83 (23.9)	1.83 (1.20, 2.79)	
No	227 (85.3)	264 (76.1)	Ref	
Neck covered				
Yes	129 (48.5)	208 (59.9)	1.59 (1.15, 2.19)	
No	137 (51.5)	139 (40.1)	Ref	
Full arm covered				
Yes	166 (62.4)	255 (73.5)	1.67 (1.18, 2.36)	
No	100 (37.6)	92 (26.5)	Ref	
Half arm covered				
Yes	100 (37.6)	88 (25.4)	0.56(0.40,0.80)	
No	166 (62.4)	259 (74.6)	Ref	
Attire outside				
*Chadder	70 (26.3)	91 (26.3)	1.44(0.95, 2.19)	
**Burqa	92 (34.6)	161 (46.5)	1.94(1.33, 2.83)	
Others	104 (39.1)	94 (27.2)	Ref	
Skin tone				
Dark	151 (59.0)	198 (59.1)	1.01(0.72, 1.42)	
Wheatish	93 (36.3)	123 (36.7)	0.89 (0.40, 1.98)	
Fair	12 (4.7)	14 (4.2)	Ref	
Season of blood draw				
Winter	158 (60.8)	176 (51.3)	1.47 (1.06, 2.04)	1.51 (1.07, 2.15)
Summer	102 (39.2)	167 (48.7)	Ref	Ref
Sun avoidance behavior				
Yes	215 (80.8)	296 (85.3)	1.38 (0.90, 2.11)	
No	51 (19.2)	51 (14.7)	Ref	
Sunlight exposure score (summer)*	26.4(75.6)	32.8 (104.7)	10.01(0.97, 10.06)	
Sunlight exposure score (winter)*	39.9 (89.6)	49.1 (121.1)	10.02 (9.98, 10.05)	

*OR* odds ratio; *CI* confidence interval

Reference: OR are compared to those women who had VDD

^*^Covering head, neck, full arms and full legs **Covering head, face, neck, full arms and full legs

Adjusted for education, BMI, parity, skin tone, sunlight exposure, attire outside. Reference: OR are compared to those women who had no vitamin D deficiency

## Discussion

In our study, 57% of women had VDD and only 24.5% of participants had optimal vitamin D status. VDD was associated with younger age, lower SES, winter season, while exercise and vitamin D supplement use were protective. A cross-sectional study among 351 patients at the out-patient department of General Medicine of a hospital in Islamabad, reported VDD present in 62.9% of females (mean age 46.03 + / − 16.18 years) and the mean vitamin D concentration was 14.09 + / − 12.93 ng/ml [13]. A study conducted in Lahore similarly reported high prevalence of VDD among healthy women of child-bearing and was also associated with low education and lack of proper sun exposure and multivitamin intake [14].

In a study conducted at an urban hospital in Boston, VDD was associated with winter season, higher body mass index, and physical inactivity [15]. In a study in Japan among 4,793 patients with rheumatoid arthritis, the mean (SD) serum 25(OH) D concentration was 16.9 ng/mL (SD 6.1), and the prevalence of vitamin D deficiency was 71.8%. Predictors of VDD were female gender, younger age, among other factors that included low serum concentrations of total protein and total cholesterol, high serum ALP concentrations, and NSAID intake [16]. VDD was also found high among pregnant women in Belgium [17]. Association of VDD with younger age among females has been consistently reported in multiple studies [18–22].

Similar to our study, VDD is found to be more common among women during winter/spring compared with summer/autumn [23–25]. In South Florida (U.S.), the mean (+ / − SD) of 25(OH)D concentration was 22.4 + / − 8.2 ng/ml in 40% of women during winter [26]. In a study in 3,327 pregnant Japanese women, VDD prevalence was 73.2% and it was higher in April after the winter season. Sun exposure of > 15 min for 1–2 days / week and usage of dietary vitamin D were protective against VDD [27]. A study conducted in Jinnah Postgraduate Medical Center, Karachi among students also showed that VDD was more common in winter [28]. Similarly, a cross-sectional study of 14,302 Chinese participants aged 18–65 years from six major cities in China reported VDD was higher among females, in spring and winter from certain residential regions [29].

A study conducted among pregnant women in Malaysia reported that intake of vitamin D was protective against VDD. However, it reported no association of VDD with age, educational, SES, employment, parity, body mass index, and sun exposure [30]. Another small cross-sectional study in Riyadh, Saudi Arabia, reported VDD among 60.2% of participants was associated with lack of usage of vitamin D, and multivitamins [31]. Another study in Riyadh, Saudi Arabia reported that absence of vitamin D supplements usage, younger age were factors associated with VDD among females [32]. VDD was 75.1% in a study in France and was associated with lack of intake of vitamin D [33]. A cross-sectional study of 634 healthy volunteers aged 18–50 years reported VDD associated with lack of multivitamin use (*p* < 0.001) [34].

Our study showing lower SES and sedentary lifestyle associated with VDD was also reported by The National Health and Nutrition Examination Survey (NHANES) 2001–2010 [35]. A cross-sectional study in Saudi Arabia, reported younger age, less exercise, less Vitamin D intake, as predictors of VDD [36].

Our study results are consistent with findings in few other studies conducted in Switzerland, China and New South Wales showing association of VDD with childbearing age, winter season and no usage of vitamin D supplement [37–39]. A large study from the Korean National Health and Nutrition Examination Survey (KNHANES) 2008–2009 among 2062 adolescents also showed that VDD was higher in senior high school students and was associated with winter season and parental vitamin D deficiency [40].

Contrary to previous studies across different ethnic backgrounds, this study within Pakistani females showed that certain factors like skin tone, obesity, sun exposure etc. were not associated with VDD. However, this lack of association between VDD and BMI was also observed in a study in Lebanon [41]. The lack of association between VDD and sun exposure is also reported in a study in Brazil, where the use of sun exposure questionnaire with 25OHD concentration showed low accuracy and its lack of discrimination between vitamin D sufficient and deficient individuals [42]. A cross-sectional study among 254 university students in Shiraz also showed no association between VDD and sun exposure[43]. The possible explanation could be that it is difficult to recall and answer to the questions related to sun exposure accurately. Moreover, there was no association found between dietary sources of vitamin D and VDD. One reason could be the low consumption of fish and milk and vitamin D fortified food items in our population.

Our study has several strengths. The study collected comprehensive information on all potential factors associated with VDD. The data was collected by trained medical officers with expertise in both medical history taking and research. The main weakness of the study is the recall bias which is inherent in any cross sectional or case control study. Intake of vitamin D supplements use was self-reported, and most of the participants were unable to recall exact dosages and durations of vitamin D usage. Another limitation of the study is that it has a limited generalizability as it was a representative sample of women coming to both private and public hospitals, but not representative of the healthy women in general population.

Public awareness and educational campaigns about the importance of adequate vitamin D concentrations, are needed with importance of sensible sunlight exposure, adequate nutritional intake of vitamin D-rich foods and vitamin D supplements to prevent adverse health outcomes related to VDD.

## Supplementary Information

**Additional File 1**. Table 1 1shows sun exposure varaibles details.

## Data Availability

The datasets used and analyzed during the current study are available from the corresponding author on request.

## Abbreviations

CI: Confidence interval
VDD: Vitamin D deficiency
OR: Odds ratio
AKU: Aga Khan University
