# Supplementary material for: Frequency and determinants of vitamin D deficiency among premenopausal and postmenopausal women in Karachi Pakistan
Source: BMC Womens Health. 2021 May 10;21:194. doi: 10.1186/s12905-021-01339-9 (PMC8108729; doi:10.1186/s12905-021-01339-9)
Supplement: Supplementary file 1 — Additional File 1. Table 1 1shows sun exposure varaibles details. [file 12905_2021_1339_MOESM1_ESM.docx]

**Supplementary File**

**Table 1.** **Weights given to sun exposure variables**

| **Variable/item** | **Weights given** |
| --- | --- |
| Part of the body exposed based on attire used outside | 1 if exposed (100% UVB absorption)  0 if covered (0 UVB absorption)  0.5 if partially covered(50% UVB absorption) |
| Use of sunscreen on different parts of body | 1 if no use (100% UVB absorption)  0.08 if sunscreen (8% UVB absorption) |
| Sun avoidance behavior | 1 if no protection practices (100% UVB absorption)  0.4 if seeking shade under trees/building etc  (40% UVB absorption) |
| Weather outdoors | 1 if sunny (100% UVB absorption)  0.5 if cloudy (50% UVB absorption)  0.75 if sunny/cloudy(75% UVB absorption) |
| Skin tone | 0.80 if Type 1 (80% UVB absorption)  0.675 if Type II (67.5% UVB absorption)  0.55 if Type III (55% UVB absorption)  0.425 if Type IV (42.5% UVB absorption)  0.30 if Type V (30% UVB absorption) |

**Tab 2. Vitamin D containing food items associated with serum 25-hydroxyvitamin in Pakistani women.**

| **Vitamin D containing Food items** | **Vitamin D> 20 ng/ml** | V**itamin D< 20 ng/ml** |  |
| --- | --- | --- | --- |
|  | **Mean (SD)** | **Mean (SD)** | **OR (95% CI)** |
| **Yogurt** | **0.19 (**0.34) | 0.23(0.45) | 1.34(0.83, 2.18) |
| **Lassi** | **0.42(**0.73) | 0.34(0.61) | 0.84(0.66, 1.08) |
| **Milk** | **0.31(**0.72) | 0.29(0.66) | 0.93(0.58, 1.47) |
| **MilkShake** | **0.77 (**1.11) | 0.69(1.19) | 0.94(0.81, 1.09) |
| **Cheese** | **0.20(**0.47) | 0.23(0.56) | 1.15(0.82, 1.61) |
| **Fish** | **0.43(**0.78) | 0.35(0.44) | 0.81(0.60, 1.10) |
| **Egg** | **0.13(**0.48) | 0.13(0.24) | 0.99(0.58, 1.71) |
| **Spinach** | **0.25(**0.34) | 0.25(0.39) | 1.21(0.41, 3.54) |
| **Fortified food items** | **0.18(**0.63) | 0.24(0.66) | 1.19(0.89, 1.58) |

**Vitamin D supplementation and Vitamin D levels**

**Tab 3. Intake of vitamin D supplements associated with serum 25-hydroxyvitamin in Pakistani women.**

|  | **Vitamin D > 20 ng/ml n(%)** | **Vitamin D < 20 ng/ml n(%)** | **OR (95%CI)** |
| --- | --- | --- | --- |
| **Vitamin D supplements** |  |  |  |
| yes | 204(76.7) | 172(49.4) | 0.30(0.21, 0.42 |
| no | 62(23.3) | 176(50.6) |  |
| **Oral Vitamin D Drops** |  |  |  |
| yes | 35(13.2) | 21(6.0) | 0.42(0.24, 0.75 |
| No | 231(86.8) | 327(94.0) |  |
| **Oral Vitamin D tabs/cap use** |  |  |  |
| Yes | 68(25.6) | 64(18.4) | 0.66(0.45, 0.97 |
| No | 198(74.4) | 284(81.6) |  |
| **Inj Vitamin D use** |  |  |  |
| Yes | 131(49.2) | 81(23.3) | 0.31(0.22, 0.44 |
| No | 135(50.8) | 267(76.7) |  |
| **Multivitamin use** |  |  |  |
| Yes | 122(45.9) | 118(33.9) | 0.61(0.44, 0.84 |
| No | 144(54.1) | 230(66.1) |  |
